# Wounds Inflicted by the Modern Rifle

**Published:** 1913-01

**Authors:** 


WOUNDS INFLICTED BY THE MODERN RIFLE. R. G. Heiner, M. D., United States Naval Hospital, Annapolis, Md. *Maryland Medical Journal,* Oct., 1912. Before the Spanish-American War it was said that the long range rifle would be a more humane instrument of warefare, as on account of the small cross section of the bullet and its high velocity it would only damage tissues actually in its path, leaving a small canal, the sides of which would fall together in good position for healing, and there would be no infection on account of the bullet being sterilized by the heat produced in its rapid flight. Consequently, if no important structures were in the path of the bullet, a rapid and complete recovery was to be expected.

Experience has proved that our modern high-power rifle is very destructive, producing far more extensive injuries than the old-style weapon.

The explosive effect caused by the high velocity was not considered, and experiment has proved that the temperature of the projectile is not raised enough to kill any germs that may be lodged on it.

The modern rifle is sighted up to 3000 yards, but is generally used at a range of under 1200 yards; although, of course, a great many wounds occur beyond that distance.

In wounds received at ranges of under 500 yards, the explosive effects are noted on all tissues; the closer the more severe. At very close ranges even muscles are pulpified, the bullet carrying with it a cone shaped mass of tissue, with its apex corresponding to the wound entrance, and its base sometimes three or four inches in diameter, corresponding to the wound of exit. Traumatic aneurisms are caused in the adjacent vessels and the shock is transmitted to the entire member. Bones are pulverized, frequently the whole shaft of a long bone; and in case of solid organs, as liver, kidneys, brain, etc., complete destruction occurs. At close range brain wounds are 100 per cent. fatal.

Between 500 and 1200 yards the wounds are more likely to be perforative, and the damage lies only in the track of the bullet; the nearer you get to 1200 yards the more clean cut the perforation.

Over 1200 yards, when the bullet loses its steadiness and begins to wobble, you have another entirely different class of wounds. The bullet still has enough velocity to perforate and is wobbling, tumbling or traveling side on, on account of which it produces extensive lacerations to soft parts and shattering of bones, but there is no explosive effect.

